# Probing the Physicochemical, Nanomorphological, and Antimicrobial Attributes of Sustainable Silk Fibroin/Copaiba Oleoresin-Loaded PVA Films for Food Packaging Applications

**DOI:** 10.3390/polym17030375

**Published:** 2025-01-30

**Authors:** Daniel S. Santos, Robert S. Matos, Erveton P. Pinto, Samuel B. Santos, Henrique D. da Fonseca Filho, Rodrigo Prioli, Irlon M. Ferreira, Tiago M. Souza

**Affiliations:** 1 Postgraduate Program in Biodiversity and Biotechnology (BIONORTE), Federal University of Amapá-UNIFAP, Macapá 68903-419, AP, Brazil; danielsousa@unifap.br; 2 Amazonian Materials Group, Physics Department, Federal University of Amapá, Macapá 68903-419, AP, Brazil; 3 Department of Physics, Federal University of Amapá, Macapá 68903-419, AP, Brazil; erveton@unifap.br; 4 Postgraduate Program in Physiological Sciences, Federal University of Sergipe, São Cristovão 49107-230, SE, Brazil; samuelbruno@gmail.com; 5 Laboratório de Desenvolvimento e Aplicações de Nanomateriais da Amazônia (LADENA), Department of Materials Physics, Federal University of Amazonas, Manaus 69067-005, AM, Brazil; hdffilho@ufam.edu.br; 6 Departamento de Física, Pontifícia Universidade Católica do Rio de Janeiro, Rio de Janeiro 22541-041, Brazil; prioli@vdg.fis.puc-rio.br; 7 Biocatalysis and Applied Organic Synthesis Laboratory, Federal University of Amapá, Macapá 68903-419, AP, Brazil; irlon.ferreira@unifap.br; 8 Department of Chemical Engineering, State University of Amapá, Macapá 68900-070, AP, Brazil

**Keywords:** PVA films, silk fibroin, copaiba oleoresin, physicochemical properties, nanomorphology, antimicrobial activity

## Abstract

We explore the development of biodegradable poly(vinyl alcohol) (PVA) films loaded with silk fibroin (SF) functionalized with copaiba oleoresin (SFCO) for potential use in active food packaging. The films were characterized, showing significant improvements in both their physicochemical and nanomorphological properties. Films containing 10% SFCO exhibited superior mechanical strength, with a Young modulus of 145 MPa and an elongation at break of 385%, compared to the control film with 42 MPa and 314%, respectively. The films also demonstrated barrier properties, with water vapor transmission rates (WVTRs) as low as 25.95 g/h·m^2^. Antimicrobial activity against *Staphylococcus aureus* and *Escherichia coli* was significantly improved, showing inhibition zones of up to 10 ± 1 mm and a minimum inhibitory concentration (MIC) of 100 µg∙mL^−1^. Three-dimensional nanomorphological analysis via atomic force microscopy (AFM) showed increased roughness in films with higher SFCO content, with root mean square (RMS) roughness values ranging from 2.70 nm to 11.5 nm. These results highlight the potential of SFCO-loaded PVA films as robust, eco-friendly alternatives to conventional packaging materials. They provide improved mechanical and antimicrobial properties, essential for extending the shelf life of perishable foods and advancing sustainability in the packaging industry.

## 1. Introduction

The food industry continually encounters challenges concerning the preservation, quality, and safety of consumer products [1]. Bioactive polymeric films have emerged as an innovative solution to these issues, functioning not only as passive packaging but also as active materials that interact beneficially with food products [2]. These films play a vital role in extending product shelf life, reducing losses, and preventing food waste while preserving the sensory attributes of food and ensuring microbiological safety [3]. In addition to preservation, these films can release bioactive compounds such as antioxidants and antimicrobials, further enhancing food safety and quality [4,5].

In recent years, bioactive products containing oil from *Copaifera* species have gained prominence due to their derivatives’ biological and pharmacological properties [6,7,8,9]. However, copaiba oil and its extracts face application challenges due to their water insolubility, chemical oxidation, and photodegradation [10,11,12,13]. To address these limitations, it is crucial to develop methods that guarantee the stability and bioavailability of bioactive compounds, such as incorporating copaiba oleoresin into polymeric matrices [14]. In this regard, *Bombyx mori* fibroin protein, derived from silkworm cocoons, has been recognized for its high biocompatibility, good mechanical behavior, adjustable biodegradability, and diverse applications in the biomedical field [15,16,17]. The molecular structure of silk fibroin demonstrates intrinsic emulsifying properties in aqueous environments. This is primarily due to its hydrophobic and hydrophilic blocks, which give fibroin an amphiphilic nature, essential for micellar structure formation [18,19,20]. Emulsification methods frequently employ copolymers and proteins as alternatives to enhance oil dispersion in aqueous vehicles [21]. In this context, it is important to highlight that silk fibroin has been suggested in the literature as a promising dispersing agent for various types of surfactants used in emulsion formulations. This is mainly due to its low production cost, minimal cellular and environmental toxicity, high biodegradability, and its status as a protein-based biomaterial [22].

Furthermore, poly(vinyl alcohol) (PVA) is widely used in the production of bioactive films due to its advantageous physicochemical and biological properties, including biodegradability, biocompatibility, and versatility [23]. PVA films offer good mechanical strength, flexibility, transparency, and an effective barrier against gasses and unwanted aromas, making them suitable for food packaging [24]. Thus, the combination of PVA films with natural oils, such as copaiba oleoresin, significantly enhances their functional properties, including antimicrobial and antioxidant capabilities, while contributing to environmental sustainability [25,26]. This approach is beneficial in the food industry, where it is essential to maintain quality and extend the shelf life of products. Incorporating natural compounds into PVA films provides an innovative and eco-friendly solution, aligning with the growing demand for sustainable and effective packaging [27].

Herein, we investigate the use of biodegradable PVA films loaded with silk fibroin functionalized with copaiba oleoresin for potential bioactive packaging. We conducted a thorough investigation into the physicochemical and nanomorphological properties of these films and their relationship with antimicrobial activity, which, to our knowledge, has not been previously reported. Our findings highlight the potential of these films as innovative, eco-friendly alternatives to traditional packaging materials, offering not only biodegradability but also superior antimicrobial properties. These attributes are essential for extending the shelf life of perishable foods, minimizing food waste, and promoting sustainability in the packaging industry. This work, therefore, provides valuable insights that could pave the way for the development of next-generation bioactive food packaging solutions.

## 2. Materials and Methods

### 2.1. Raw Materials

We used silkworm cocoons from Bombyx mori L. and purchased hydrolyzed poly(vinyl alcohol) (PVA, 89,000–98,000 g/mol, >99%) from Sigma-Aldrich (São Paulo, Brazil). *Copaifera officinalis* oleoresin (CO) was from Ferquima Indústria e Comércio Ltda. (Vargem Grande Paulista, São Paulo, Brazil). The materials and reagents were of analytical grade and used as received without further purification. All experiments were conducted using distilled or deionized water.

### 2.2. Silk Fibroin (SF) Extraction

The silk fibroin was obtained following a previous protocol [22]. In a typical procedure, silkworm cocoons (3 g) were cut and immersed in 500 mL of a 2% Na_2_CO_3_ solution. The mixture was stirred and heated at 100 °C for 30 min to produce fibrous material. The fibers were rinsed with 1000 mL of distilled water, dried at 70 °C for 24 h, and then dissolved by adding 50 mL of a solution comprising H_2_O:EtOH in a ratio of 8:2:1 M at 80 °C with stirring for 6 h. The solution was dialyzed at room temperature for 3 days, with the water being replaced every 24 h. After dialysis, the solution was centrifuged at 75× *g* for 10 min to remove impurities and stored at a 2% (*v*/*v*) concentration at 10 °C.

### 2.3. Preparation of the Bioactivated SFCO Emulsion

The SFCO emulsion was prepared using a prior method [20], resulting in a total volume of 10 mL. The formulation consisted of 25% SF suspension, 5% CO, 25% isopropanol, and 45% distilled water. The CO and isopropanol were combined in a 15 mL beaker and stirred at 300 rpm for 3 min. Next, the aqueous phase, containing SF and distilled water, was gradually added at 0.5 mL/min while stirring continuously for 60 min. The final emulsion was then stored at 18 °C and labeled as the silk fibroin–copaiba oleoresin (SFCO) formulation.

### 2.4. Preparation of the Films

Initially, PVA was dispersed in 200 mL of deionized water. The suspension was stirred at 300 rpm and maintained in a water bath at 90 °C for 1 h to obtain 5% and 10% (*w*/*v*) solutions. After gelation, the solution was stirred until it cooled to 40 °C. The SFCO formulations were added at varying concentrations, as shown in Table 1, and stirred for 30 min until fully homogenized. Finally, 20 g of the film-forming solution was evenly spread onto Petri dishes and dried for 24 h at 40 °C in a digital air circulation oven.

### 2.5. Physicochemical Characterization

The ultraviolet–visible (UV-vis) spectra of SFCO formulations and films were recorded with a UV-vis spectrophotometer (BEL PHOTONICS UV–M51) across a spectral range of 190–800 nm. The surface microstructure was analyzed using a scanning electron microscope (SEM) (TM3030Plus Tabletop Microscope, Hitachi, Japan) operating at 5 kV. The porosity (pore area fraction) of the SFCO films was determined from SEM images using FIJI ImageJ freeware software, version 1.53t [28]. Hygroscopicity and water solubility were determined using a previous protocol [29]. Film samples (0.02 m × 0.02 m) were cut and weighed to obtain dry mass (W_1_). They were then immersed in 50 mL of deionized water for 24 h at room temperature. After removal, surface water was blotted off and the wet films were weighed (W_2_). The samples were then dried at 45 °C for 24 h and reweighed (W_3_). Each sample was tested in triplicate, with average values calculated for water absorption (H) and solubility (S) using Equations (1) and (2).

(1) $$\%H=\left[\frac{W_{2}-W_{3}}{W_{3}}\right]\times 100$$

(2) $$\%S=\left[\frac{W_{1}-W_{3}}{W_{1}}\right]\times 100$$

The water vapor transmission rate (WVTR) was determined gravimetrically at 25 °C, following the ASTM E96/E96–13 standard [30] minor modifications, as reported in [31]. Films were fixed to the openings of glass vials with minor modifications, as reported in [31]. Films were fixed to the openings of glass vials (6 mm diameter) containing 1 mL of distilled water. The setups were weighed and placed in a desiccator with blue silica gel, maintained at 25 °C and 50% relative humidity (H). Mass measurements (g) were recorded every 24 h for 9 days. The variation in mass was plotted against time (t) and analyzed using linear regression. The slope (g/t) was determined by the loss of mass in grams as a function of time in h and WVTR was calculated using Equation (3), where A is the permeation area (m^2^).

(3) $$WVTR=\left(g/t\right)/A$$

We conducted a tensile test following the ASTM Standard D882-02 [32], with minor modifications. We evaluated the elongation at break (%) and the modulus of elasticity (MPa) for all samples. For this purpose, rectangular specimens measuring 10 × 75 mm were prepared and tested at room temperature using an EMIC DL 30,000 universal testing machine (Brazil). The testing setup included a 500 N load cell, an initial grip separation of 25 mm, and a deformation rate of 10 mm/min.

We further analyzed the films’ adhesion, deformation, and elasticity properties using an atomic force microscope (AFM), model NX-10 (Park Systems Corp., headquartered in Suwon, South Korea). In PinPoint mode, we acquired topographic images and force curves for each image’s pixel, with 1024 data points per curve. These curves, recorded at a tip approach/retraction speed of 10.0 µm/s, allowed us to assess hardness, deformation, and adhesion. We estimated the elastic modulus using the Hertz model with a tip radius of 10 nm and a Poisson’s ratio of 0.26. Measurements were taken in three regions per sample, scanning 18 × 18 μm areas at 256 × 256 pixels, with a pixel size of 70.3 nm and a scan rate of 1 Hz. The reported adhesion force, deformation, and elastic modulus values were averaged from all measurements.

Finally, the wettability of the films was evaluated using a goniometer (model 590 F3, Ramé-Hart Instrument Co., Succasunna, NJ, USA) under ambient conditions (66.8 ± 1.3% relative humidity, 26.0 ± 0.3 °C). Film samples with an area of 2.0 cm^2^ were secured in the sample holder using double-sided tape. Subsequently, 8 μL of deionized water was applied to their surfaces. The contact angles were measured immediately after the drop application, capturing 100 images (at 0.0001 s each) and averaging the left and right angles to provide 100 measurements per sample.

### 2.6. Morphological and Spatial Analysis

A thorough and detailed analysis of the films’ topography was conducted using AFM topographic maps. These maps were acquired in the AFM operating in non-contact mode using a Tap300Al-G tip (NanoAndMore USA Inc., Watsonville, CA, USA) with a resonance frequency of 300 kHz and a spring constant of 40 N/m. This study was performed at room temperature and the scan rate for image acquisition was 0.5 Hz. The process provided a data matrix for each scan with 512 × 512 pixels, where each pixel represents a recorded height value. All measurements were performed using three different randomly selected regions (30 μm × 30 μm) of the AFM images. The results are presented as mean ± standard error.

We analyzed the roughness of SFCO-loaded PVA film surfaces using the root mean square (RMS) parameter, which in practice is the standard deviation of the surface’s heights. This parameter was computed using Gwyddion 2.56 [33], according to Equation (4):

(4) $$Roughness=\sqrt{\frac{1}{N}\sum_{n=1}^{N}{\left(Z_{n}-\bar{Z}\right)}^{2}}$$

where N is the total number of pixels, $Z_{n}$ is a measured height value, and $\bar{Z}$ is the mean height of set.

To evaluate the uniformity of the height’s histogram, we computed the topographic entropy using Equation (5) with a binless approach, as has been previously described in the existing literature [34].

(5) $$E_{T}=\frac{\Lambda^{\left(2\right)}-{\Lambda_{min}}^{\left(2\right)}}{{\Lambda_{max}}^{\left(2\right)}-{\Lambda_{min}}^{\left(2\right)}}$$

where ${\Lambda_{min}}^{\left(2\right)}$ and ${\Lambda_{max}}^{\left(2\right)}$ were used for normalization (i.e., limiting $E_{T}$ between 0 and 1), aiming for a reliable comparison between different samples. The higher the E_T_ value, the more uniform the surface, with E_T_ = 1 indicating perfect uniformity. Furthermore, $\Lambda^{\left(2\right)}$ is the Shannon-based entropy that was calculated according to Equation (6).

(6) $$\Lambda^{\left(2\right)}=-\sum_{i=1}^{N}\sum_{j=1}^{N}p_{ij}\cdot \log\left(p_{ij}\right)$$

where $p_{ij}$ is the probability of each height value of the matrix not being an outlier in the height distribution and N is the total number of pixels.

The persistence of spatial patterns on the surface of the films was evaluated using the autocorrelation functions (ACFs). An ACF applied to AFM images measures the relationship of a height value with a value before it. For AFM data, the one-dimensional ACFs based on scanning profiles along the *X*-axis can be evaluated according to Equation (7) [35].

(7) $$G_{x}\left(\tau \right)=\frac{1}{N(M-m)}\sum_{l=1}^{N}\sum_{j=1}^{M-m}z_{j+m,l}\cdot z_{j,l}$$

where z, N, and M represent the height, the number of rows, and the number of columns, respectively. Furthermore, by setting τ = m Δx, the function can be evaluated in a discrete set of τ, where Δx represents the distance between two points on the *X*-axis. The lateral correlation length (L) was calculated by fitting G_x_ to a Gaussian function, according to Equation (8) [35]. Height values separated by a distance less than or equal to L tend to have the same behavior in relation to the average height, that is, peaks followed by peaks or valleys followed by valleys. The higher the value of L, the greater the long-range persistence of the topography.

(8) $$G_{x}\left(\tau \right)=\sigma^{2}exp\left(\frac{-\tau }{L}\right)$$

Our spatial analysis was enriched using Moran’s index (I), which was recently introduced by Pinto et al. [36], further enhancing the spatial autocorrelation analysis of AFM images. The Moran index is a standardized measure of spatial autocorrelation among variables of neighboring areas and was determined according to Equation (9).

(9) $$I=\frac{N}{\sum_{i}\sum_{j}W_{ij}}\frac{\sum_{i}\sum_{j}W_{ij}\cdot \left(Z_{i}-\mu \right)\cdot (Z_{j}-\mu )}{\sum_{i}{\left(Z_{i}-\mu \right)}^{2}}$$

where N is the total number of areas; $z_{i}$ and $z_{j}$ are the height values of areas i and j, respectively; $w_{ij}$ is the spacial weight (1 for connected areas and 0 otherwise, according to the queen-type neighborhood criterion [36]); and μ is the average height of the image. Moran correlograms were generated using the *sp.correlogram* function from the spdep package written in R language using RStudio freeware software, version 2024.12.0+467 [37]. The Moran correlogram provides insights into the short-range correlation between surface heights, which can identify the presence of spatial clusters.

To expand our analysis of the film morphology, we captured detailed topographical information using Minkowski functionals (MFs). These functionals are metrics used to examine 2D or 3D surface variations from an AFM topographical map [38]. They are valuable for providing insights into the surface features at micro- or nanoscale levels. Utilizing Gwyddion freeware software, version 2.67 [33,39], we calculated three Minkowski functionals: volume (V), boundary (S), and connectivity (χ). To measure the Minkowski functionals, the images were binarized using different height values as thresholds. Heights below the threshold are called black pixels and otherwise white pixels. Then, the functionals can be plotted as functions of this threshold. Minkowski functionals are defined by Equations (10)–(12) [33], with N_white_ representing the number of white pixels, N the total number of pixels, N_bound_ the number of white–black pixel boundaries (pixels equal to the threshold), and C_white_ and C_black_ the number of white and black clusters, respectively.

(10) $$V=\frac{N_{white}}{N}$$

(11) $$S=\frac{N_{bound}}{N}$$

(12) $$\chi =\frac{C_{white}-C_{black}}{N}$$

### 2.7. Antimicrobial Activity

Antimicrobial activity was evaluated using the Mueller–Hinton agar diffusion method (Mueller–Hinton CLSI, 2005) [40], conducted in triplicate for each microorganism, utilizing standard strains of *Staphylococcus aureus* ATCC 25923 and *Escherichia coli* ATCC 25922. Cultures were prepared by incubating overnight at 35 ± 2 °C in 5 mL of Brain Heart Infusion (BHI) broth and the suspension was adjusted to a 0.5 McFarland standard. Subsequently, 100 µL of the adjusted suspension was inoculated onto Petri dishes containing Mueller–Hinton agar (pH 7.2–7.4), which had been previously solidified using a Drigalsky loop. Film disks of 0.8 cm^2^ were then placed on the surface of the inoculated medium. The film constituents served as negative controls, while gentamicin at 50 µg/mL was used as a positive control due to its known antimicrobial activity against standard ATCC strains. Antimicrobial activity was determined by measuring the inhibition zones in mm. The minimum inhibitory concentration (MIC) was evaluated in triplicate for each antimicrobial-active formulation sample using the broth microdilution method according to the guidelines of the National Committee for Clinical Laboratory Standards (NCCLS). A 96-well ELISA plate was prepared by adding 100 µL of Mueller–Hinton broth to each well, followed by 50 µL of the samples at a concentration of 50 µg/mL, in addition to 50 µL of the bacterial inoculum. Negative controls were performed with wells containing only culture medium to check for contamination, positive controls with wells containing inoculum and culture medium to assess the viability of the tested strains, and additional controls with wells containing culture medium, dimethyl sulfoxide (DMSO), and bacterial suspension to ensure that DMSO did not inhibit bacterial growth. After incubating the microplate for 24 h at 35 ± 2 °C, a 0.01% aqueous resazurin solution was added to each well. The microplate was incubated for an additional 1 h and the reading was performed visually, where blue coloration indicated the absence of bacterial growth and pink/red coloration indicated microbial metabolism.

### 2.8. Statistical Analysis

The quantitative results were expressed as means and standard error, organized, according to relevance, in tables, graphs, charts, and figures. Significant differences between results were evaluated using one-way analysis of variance (ANOVA) and Tukey’s pairwise test (*p* < 0.05) in the PAST software, version 4.03 [41].

## 3. Results and Discussion

### 3.1. Physicochemical Aspects

**Figure 1:**
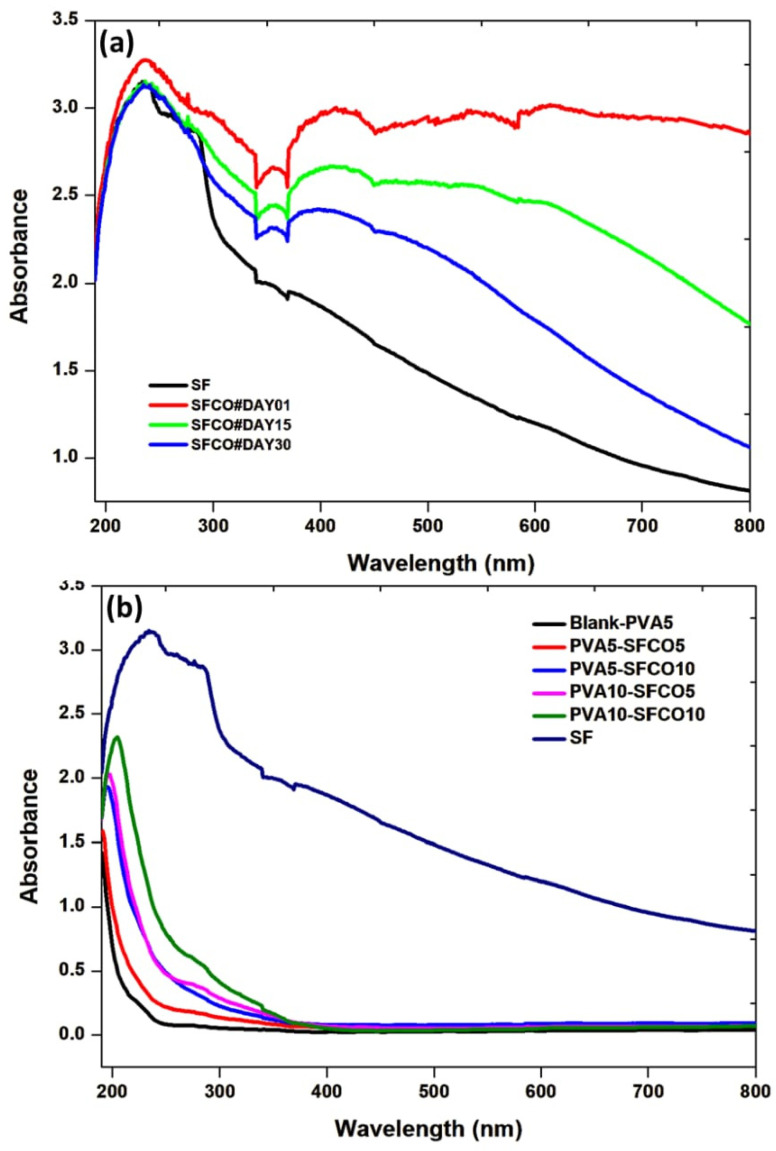
UV-vis spectra: (**a**) pure SF and SF-CO emulsions and (**b**) SF/CO-loaded PVA films.

The stability of SFCO emulsions and the optical absorption of SFCO-loaded PVA films were investigated using UV-Vis spectroscopy, with the spectra shown in Figure 1. Figure 1a shows a broad absorption band at 230–300 nm for both SF and SF-CO emulsions, indicating the presence of aromatic amino acids like tyrosine, phenylalanine, and tryptophan in SF molecular chains [42,43]. Optical absorption below 250 nm is primarily due to the peptide (amide) bonds in the SF main chain. In contrast, the absorption band near 280 nm originates from the side chains of silk fibroin [44]. Notably, significant changes in absorbance are observed in the 370–450 nm range and around 580 nm for SFCO formulations compared to pure SF. Absorption values are highest on the 1st day but decrease on the 15th and 30th days. This trend suggests a decrease or photodegradation of the components in copaíba oil. According to Nogueira et al. [45], the intense absorption below 400 nm may indicate the presence of α–tocopherol and fatty acids, such as palmitic and oleic acids, which absorb below 375 nm. The findings indicate that the emulsions should be used within the 1st and 15th day after synthesis to retain the functional properties of copaíba oil. Similarly, changes in the absorption spectra of PVA films containing SFCO emulsions suggest the formation of new photoproducts. Figure 1b shows an absorption band around 200 nm for all films, which can be attributed to the copaíba oil terpenes. In particular, the peak at 205 nm for PVA5–SFCO5 suggests β-caryophyllene in the polymeric matrix. Thus, the films with higher absorbance {PVA5–SFCO10, PVA10–SFCO5, and PVA10–SFCO10} are expected to be slightly more translucent than those with lower absorbance {Blank–PVA5 and PVA5–SFCO5}.

**Figure 2:**
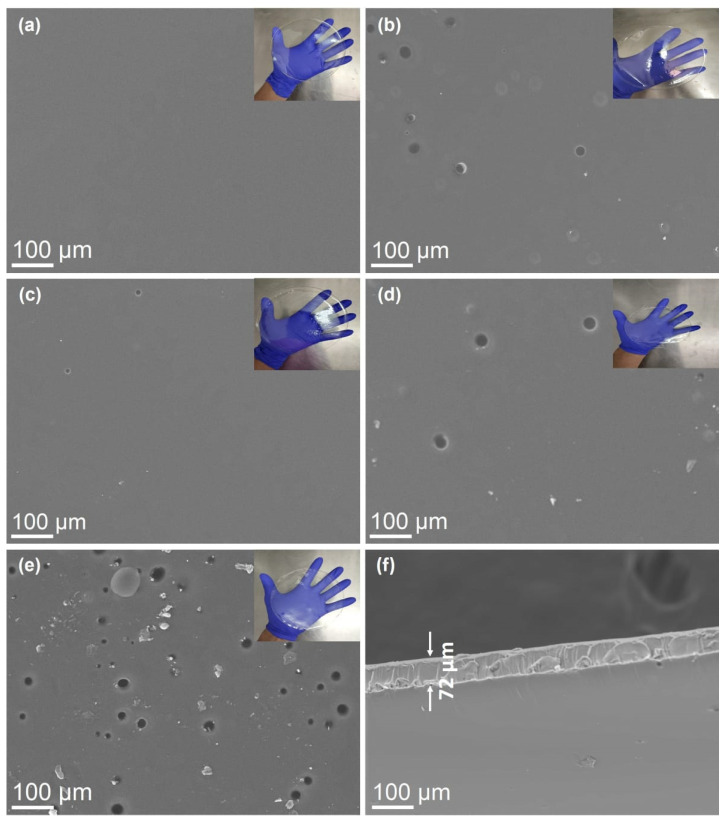
SEM images: (**a**) Blank–PVA5, (**b**) PVA5–SFCO5, (**c**) PVA5–SFCO10, (**d**) PVA10–SFCO5, and (**e**) PVA10–SFCO10. (**f**) Representative SEM cross-section image of Blank–PVA5.

The surface morphology of pure and SF/CO-loaded PVA films is shown in Figure 2. The inset figures in Figure 2a–e show that all films exhibit transparent features. The SEM image of the unloaded film (Figure 2a) shows a smooth and homogeneous surface, with no visible grains or distinct morphological artifacts. However, the samples loaded with different concentrations of SF/CO and PVA {PVA5–SFCO5, PVA5–SFCO10, PVA10–SFCO5, and PVA10–SFCO10} (Figure 1b–e) display porous structures with diameters ranging from 20 to 45 µm. The PVA10–SFCO10 film exhibited the highest porosity (~1.8%), while the PVA5–SFCO10 film showed the lowest porosity (~0.05%). The other samples presented intermediate values, with PVA10–SFCO5 at ~0.4% and PVA5–SFCO5 at ~0.6%. Further, the cross-section image of the unloaded film (Figure 2f) shows that the samples have an average thickness of 72 µm.

The variation in the pore diameter, which increases with the concentration of SF/CO, indicates that the pore size is dependent on the SF/CO ratio. Notably, pore formation is more prominent in films with higher concentrations of SF/CO. This was also observed for the surface morphology of different PVA–starch films incorporated with lemongrass oil [46]. This occurrence is largely due to the critical role of SF in stabilizing emulsions, which affects the final structure and functional properties of the films. This is further supported by previous studies [47,48,49], which have extensively demonstrated that the porous structure seen on the surfaces of polymeric films functionalized with vegetable oils is linked to the evaporation of oil droplets during the drying process.

A very porous surface may be an indication that a significant amount of oil has migrated to the film surface and volatilized, leaving holes. This behavior negatively affects the oil retention efficiency and may decrease the antimicrobial activity of the sample. On the other hand, a discontinuous structure can influence the mechanical properties of the material, since the holes can deform and dissipate energy during mechanical stress.

The mechanical and interface properties of the films are presented in Table 2. All formulations resulted in hydrophilic films (contact angle less than 90°), with hydrophilicity positively affected by the incorporation of SF-CO. On the other hand, the hygroscopicity, water vapor transmission rate, and solubility of the films decreased with the incorporation of SF-CO, which appears to be consistent with the hydrophobic characteristic of the active ingredient. However, it is important to emphasize that silk fibroin is a natural surfactant capable of enhancing interaction with water due to the polar tail of its amphiphilic molecule, which likely contributed to the positive effect on hydrophilicity.

The stiffness of the films increased with the presence of SF-CO in the polymer matrix, as Young’s modulus was higher for all formulations containing the active solution compared to the control (Blank–PVA5), suggesting that silk fibroin may have acted as a crosslinking agent. On the other hand, the increase in elongation at break was attributed to the deformation of the pores left by the volatilization of the oil droplets.

**Figure 3:**
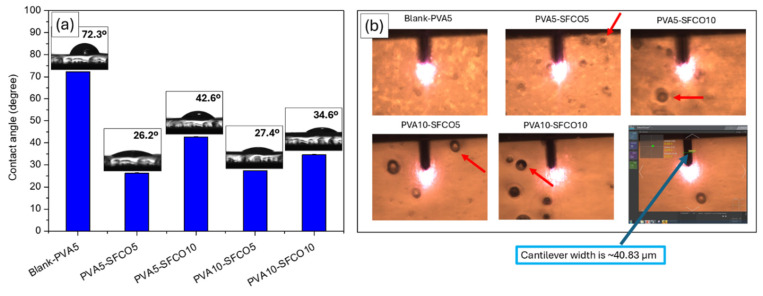
Wettability and optical microscopy: (**a**) contact angle measurements and (**b**) images captured with an optical microscope coupled to the AFM. Red arrows highlight structures associated with microdroplets of copaiba oil.

For a more comprehensive discussion of the wettability of the samples, Figure 3a shows the water droplets scattered over the surface of the films. Previous studies have similarly documented that the addition of silk fibroin enhances the wettability of polymeric films [50,51,52]. Figure 3b displays images of the film surface obtained using an optical microscope coupled with an AFM. These images show spherical structures with diameters exceeding 40 μm in films containing SF-CO, which are likely oil microdroplets, corroborating with the SEM images.

**Figure 4:**
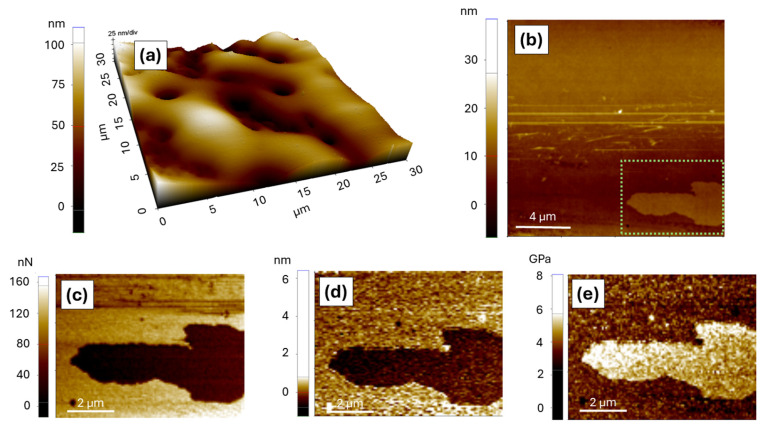
Local surface characteristics: (**a**) 3D representation of oil coverage, (**b**) region with PVA crystal (identified by the green box), (**c**) adhesion map on the PVA crystal (scale in nanonewton, nN), (**d**) deformation map on the PVA crystal (scale in nanometer, nm), and (**e**) elasticity map (elastic modulus) on the PVA crystal (scale in gigapascals, GPa).

Furthermore, the AFM images acquired in non-contact mode in regions between the microdroplets of the SFCO-containing films revealed areas with spread oil (Figure 4a) and small crystals (Figure 4b), likely crystallized or non-solubilized PVA from the film synthesis. In this regard, Sau et al. [53] observed a similar behavior for PVA films prepared from a film-forming solution homogenized at 90 °C, which presented a crystallinity of about 81.8%.

Figure 4c–e show the adhesion, deformation, and elasticity maps of the PVA crystal. The contrast in these maps indicates that these structures are less adhesive and more resistant compared to the surrounding material, supporting their identification as crystallized PVA. Adhesion, deformation, and elastic modulus measurements from the maps were acquired, with average values presented in Table 3. The macroscopic adhesiveness of the films reflects the combined effects of microdroplets with SFCO interfaces and the physical properties observed between microdroplets (spread oil, PVA crystals, and topography).

### 3.2. Nanomorphological and Spatial Analysis

**Figure 5:**
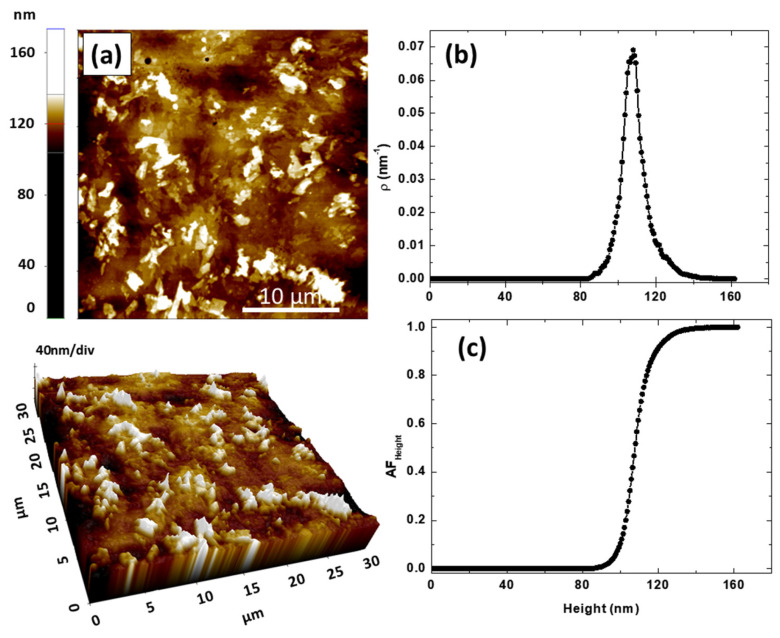
Topography of the control film (Blank–PVA5): (**a**) 2D and 3D AFM micrographs, (**b**) height distribution density, and (**c**) Abbott–Firestone curve.

Figure 5 provides an analysis of the surface morphology and height distribution of the pure PVA sample (Blank–PVA5). In Figure 5a, both 2D and 3D AFM micrographs are shown. The 2D image shows the surface topology with height variations ranging up to 163 nm, indicated by a color gradient, where darker regions represent lower heights and brighter regions correspond to higher elevations.

The 3D micrograph provides a vivid representation of the sample’s surface texture, with the *x* and *y* dimensions spanning 30 µm each and the *z*-axis indicating notable surface roughness. This observation is further supported by an average RMS roughness value of 9.14 nm (Table 4). Figure 5b shows the height distribution density of the sample’s surface. The peak at approximately 110 nm suggests a prevalent surface height, with the majority of surface points clustering around this value, indicating a relatively uniform height distribution with minimal outliers, which was also confirmed by the topographic entropy value greater than 0.9 (Table 4).

Figure 5c features the Abbott–Firestone curve, also known as the bearing area curve, which is a cumulative distribution function of surface heights [54,55]. The Abbott–Firestone curve is instrumental in tribology and surface engineering as it provides insights into the functional properties of the surface, such as load-bearing capacity and surface contact area [56]. The curve shows a steep rise around 80 to 120 nm, indicating that most of the surface area is within this height range, confirming the findings from the height distribution curve. This rapid transition implies that a significant portion of the surface is at or near the peak height, which may influence the sample’s interaction with other surfaces or materials.

**Figure 6:**
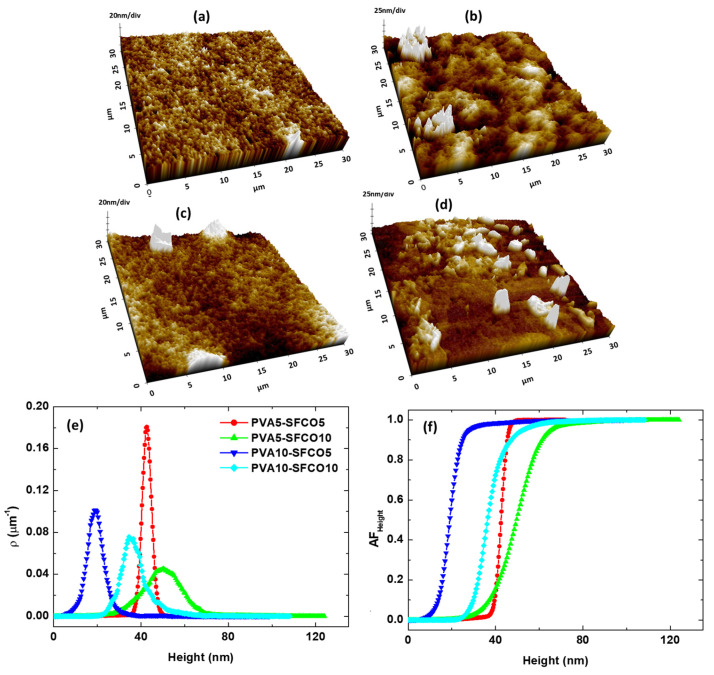
Topographic analysis of the samples: (**a**–**d**) 3D AFM micrographs of PVA5–SFCO5, PVA5–SFCO10, PVA10–SFCO5, and PVA10–SFCO10, respectively; (**e**) height distribution density; and (**f**) Abbott–Firestone curve of the height (AF_Height_).

Figure 6 illustrates a comparative analysis of the surface morphology of PVA-based films with different combinations and SFCO contents. The 3D AFM images in Figure 6a–d showcase the topographies of PVA5–SFCO5, PVA5–SFCO10, PVA10–SFCO5, and PVA10–SFCO10 samples, respectively. These images reveal notable differences in surface roughness and morphology across the samples. In Figure 6a, the PVA5–SFCO5 sample exhibits a relatively smooth surface with height variations up to 67 nm. This smoothness, further confirmed by the computed average RMS roughness (2.7 nm, Table 4), is indicative of a homogeneous dispersion of SF within the PVA matrix. Figure 6b shows the PVA5–SFCO10 sample, which displays a more textured surface with prominent peaks reaching 120 nm. This sample has an average RMS roughness of 11.52 nm (Table 4), which is a notable increase compared to the sample PVA5–SFCO5, indicating that a higher concentration of SF-CO increases the RMS roughness of PVA films.

In Figure 6c, the PVA10–SFCO5 sample shows surface height variations of up to 99 nm, with an average RMS roughness of 5.73 nm (Table 4). Increasing the PVA concentration to 10% while maintaining the SF content at 5% results in a rougher surface compared to the PVA5–SFCO5 sample, but it is still smoother than the PVA5–SFCO10 sample. This indicates that the balance between PVA and SF-CO content plays an important role in determining the surface characteristics. Figure 6d shows the PVA10–SFCO10 film, which presented height variations up to 109 nm and an average RMS roughness of 9.10 nm (Table 4), and low uniformity confirmed by the topographical entropy value of 0.872 (Table 4). This sample displays a greater presence of PVA crystals due to the increased polymer concentration, which negatively affects the surface uniformity.

Figure 6e shows the samples’ height distribution density curves. The PVA5–SFCO5 sample displays a narrow height distribution peak around 45 nm, indicating a uniform surface height, similar to the Blank–PVA5 sample. In contrast, the PVA5–SFCO10 sample shows a broader distribution with a peak around 55 nm, which reflects increased surface roughness due to a higher SF-CO content. The PVA10–SFCO5 and PVA10–SFCO10 samples show narrower distributions than the PVA5–SFCO10 film, with peaks around 19 nm and 35 nm, respectively. This highlights the compounded effect of higher PVA and SFCO concentrations on surface roughness, corroborating with the RMS roughness values (Table 4). The topographic entropy values in Table 4 quantitatively complement these observations, since all samples showed pronounced uniformity in the height distribution, with E_T_ values greater than 0.8.

The Abbott–Firestone curves presented in Figure 6f provide additional insight into the surface characteristics. The PVA5–SFCO5 sample shows a steep transition, similar to the pure sample, indicating that a large fraction of the surface area is concentrated around the peak height. The PVA10–SFCO5 sample’s curve is less steep, suggesting a more gradual increase in surface height. The PVA5–SFCO10 and PVA10–SFCO10 samples exhibit the least steep curves, reflecting the highest surface roughness and the most heterogeneous surface features among all of the samples.

**Figure 7:**
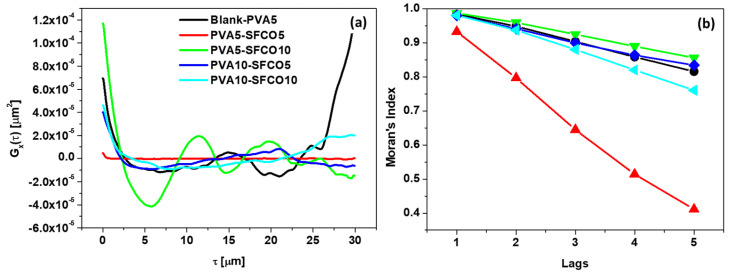
Spatial analysis of the samples: (**a**) autocorrelation function and (**b**) Moran correlogram.

Figure 7 displays two important tools used in our spatial analysis of the surface: the autocorrelation function (ACF) in Figure 7a and the Moran correlogram in Figure 7b. The ACF curves show oscillatory behavior, indicating periodicity in the spatial distribution of heights on the surface. Despite this, the PVA5–SFCO10 sample (represented by the green line) shows the most prominent oscillations, suggesting well-defined periodic features. In contrast, the other samples show less pronounced oscillations, which may indicate more randomized surface structures or less well-defined periodicity, which is remarkable for the PVA5–SFCO5 sample.

Unlike the autocorrelation function, the Moran index assesses the correlation not between individual points, but rather between neighboring areas. This index is a measure of short-range correlation, and its correlogram allows us to infer the long-range correlation by examining how the Moran index decays with the neighborhood lag. Thus, we observe that the PVA5–SFCO5 film presents a lower long-range correlation and a reduced tendency to cluster. This indicates that the film is predominantly influenced by higher spatial frequencies, which agrees with its low lateral correlation length of 0.40 μm (Table 4). According to Zhang and Sundararajan [57], a smaller lateral correlation length suggests a larger real contact area, which favors interface interactions. The other samples did not show significant differences concerning the spatial autocorrelation of heights on the surface.

**Figure 8:**
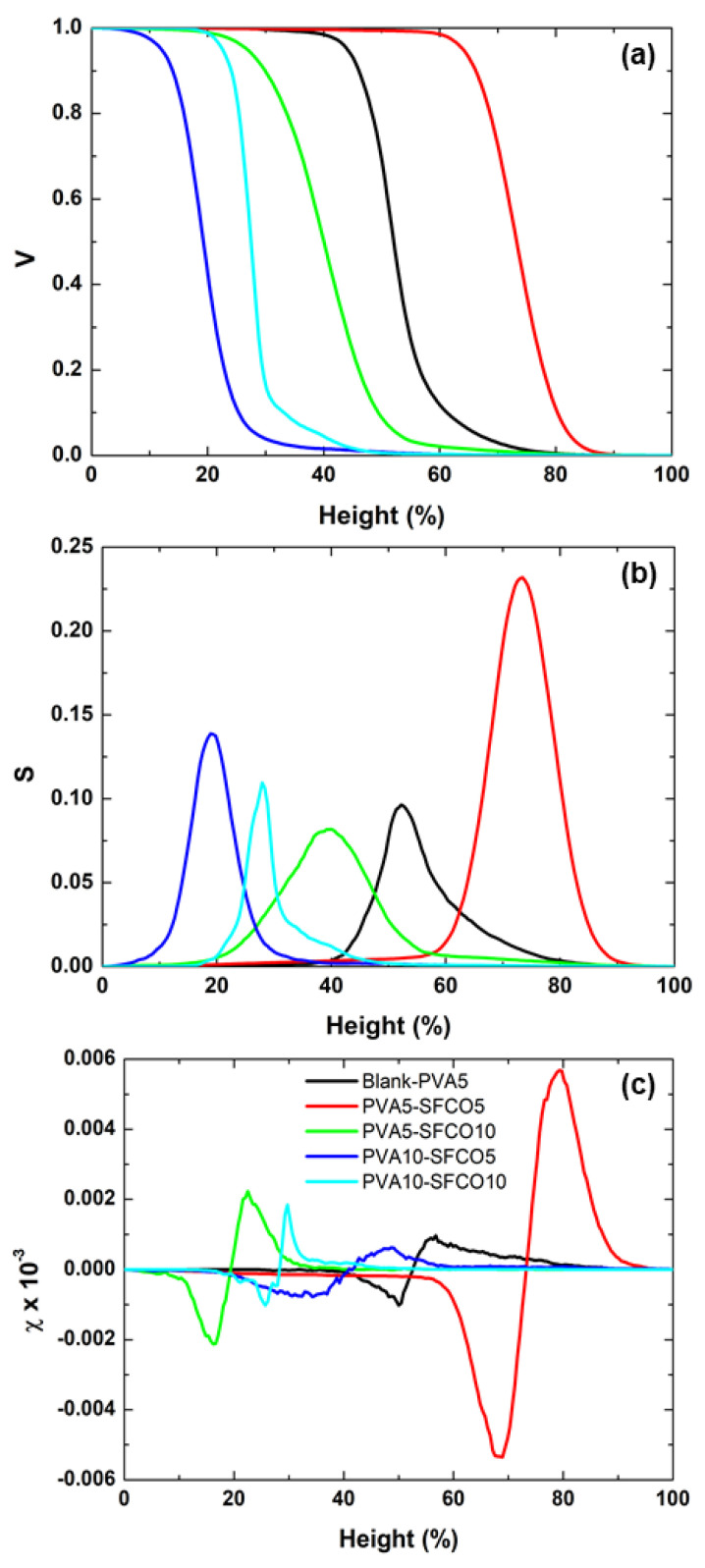
Minkowski functionals of the samples: (**a**) volume—V, (**b**) boundary—S, and (**c**) connectivity—χ.

In Figure 8, we have the Minkowski functionals (MFs) for a more comprehensive analysis of the mass distribution and connectivity in 3D. The Minkowski volume (V) represents the total space occupied by a structure, quantifying the overall size or material amount. The Minkowski boundary (S) measures the surface length in 3D, reflecting the interface complexity between a geometric feature and its surroundings; a larger value indicates a more intricate surface. The Minkowski connectivity (χ) or Euler characteristic quantifies structural connectivity, indicating isolated parts and holes, i.e., it describes connectedness and the presence of cavities or tunnels in a 3D structure [58,59,60].

As seen in Figure 8a, the PVA5–SFCO5 sample concentrates a smaller amount of matter in the peaks, while the PVA10–SFCO5 sample shows the opposite behavior. Thus, although the correlation length favors the real contact area for the PVA5–SFCO5 film, the reduced concentration of matter in the peaks makes these samples less interactive. Furthermore, Figure 8b shows that the PVA5–SFCO5 film is the most complex with a greater dominance of pointed regions (larger peak for S). In contrast, the PVA5–SFCO10 sample has characteristics of a surface dominated by flattened and smooth regions (smaller peak for S). Figure 8c again highlights the PVA5–SFCO5 and PVA10–SFCO5 films, since the first one presents the highest minimum and maximum values for the connectivity curve, suggesting high connectivity between the structures that compose its surface, and the second one shows the opposite behavior. A more pronounced minimum value in the connectivity curve indicates a greater number of cavities on the surface.

In summary, for a surface with a protective function, the ideal approach would be to minimize the interaction sites with the external environment, that is, to reduce the real contact area and, consequently, minimize the surface energy. To achieve this, the surface should exhibit a large lateral correlation length, minimal material at the peaks, low roughness, large clusters, a dominance of flattened structures, and low spatial frequencies. Thus, the PVA5–SFCO10 and PVA10–SFCO5 films were the most promising concerning their surface morphological characteristics.

### 3.3. Antimicrobial Assay

Typically, antimicrobial packaging materials are prepared using inorganic, organic, and biologically active substances [61]. To address health and environmental concerns, there is a growing preference for natural, effective, and non-toxic antimicrobial agents. Essential oils (EOs), which are plant-derived secondary metabolites with potent antimicrobial properties against various foodborne pathogens, have become a common choice [62]. The antimicrobial activity of the samples against *Staphylococcus aureus* (*S. aureus*) and *Escherichia coli* (*E. coli*) is displayed in Table 5, showcasing their effectiveness in inhibiting microbial growth. The analysis highlights the comparative performance of these samples, including formulations combining silk fibroin and copaiba oil (SFCO) with polyvinyl alcohol (PVA), along with control samples like gentamicin and standalone copaiba oil (CO). Gentamicin, a well-known antibiotic used for probing antimicrobial properties of materials [63,64], serves as the positive control, exhibiting the highest inhibition zones, 16 ± 2 mm against *S. aureus* and 15 ± 2 mm against *E. coli*. This result underscores its potent antimicrobial efficacy, setting a benchmark for evaluating other formulations.

CO demonstrated the most significant antimicrobial activity among the samples compared to the positive control, forming inhibition zones of 14 ± 1 mm for *S. aureus* and 12 ± 1 mm for *E. coli*, with no significant difference observed between them. In this regard, CO is known for its broad-spectrum antimicrobial properties, which are attributed to its rich composition of terpenes and sesquiterpenes [65,66]. The SFCO formulation, which combines silk fibroin with copaiba oil, also demonstrated notable antimicrobial activity, with inhibition zones of 12 ± 1 mm against *S. aureus* and 10 ± 1 mm against *E. coli*. In this case, the efficacy compared to pure copaiba oil may be attributed to the interaction between silk fibroin and copaiba oil, which could affect the release rate of the active compounds from the formulation.

PVA-based films loaded with SF-CO showed no significant differences in their antimicrobial activity when varying the concentration of SF-CO and the amount of PVA used. The antimicrobial activity observed for the PVA-SFCO films ranged from 8 ± 1 mm to 10 ± 1 mm against *S. aureus* and 8 ± 1 mm against *E. coli*. However, it is important to highlight that the PVA10–SFCO5 sample statistically exhibited a similar effect to the SF-CO formulation against *S. aureus*. Similarly, all PVA-SFCO film samples demonstrated antimicrobial effects with no significant differences compared to the SF-CO formulation against *E. coli.* Thus, our results demonstrate that the PVA-based films can be tailored for specific applications by adjusting the concentrations of PVA and the active antimicrobial agents. For instance, films with higher PVA content may be appropriate to applications where mechanical strength and durability are critical, while films with higher SFCO content may be preferable for applications requiring just antimicrobial protection.

The minimum inhibitory concentration (MIC) results revealed critical insights into their efficacy compared to pure samples. The MIC for all film samples was uniformly determined to be 100 ppm, the lowest concentration showing a growth inhibition halo for both *S. aureus* and *E. coli* [67]. Table 6 illustrates the MIC values for the different film samples and compares them with those of pure CO and SFCO. For the *S. aureus* strain, the film samples exhibited an MIC of 100 ppm across the board. This indicates that the films have consistent antimicrobial activity, though it is less effective than the pure substances. Pure copaiba oil and SFCO achieved superior antimicrobial results, with MIC values of 50 ppm for both *S. aureus* and *E. coli* (for CO), 50 ppm for *S. aureus*, and 100 ppm for *E. coli* (for SFCO). Notably, the lower MIC values for the pure samples indicate that copaiba oil and SFCO have higher antimicrobial efficacy compared to their concentrations within the films. This is because the films contain SFCO at lower concentrations compared to the pure samples, which likely contributes to their reduced antimicrobial effectiveness. However, these results demonstrate that the films exhibit notable antimicrobial activity, especially given their lower concentration of the raw active substances SF and CO. This suggests that the antimicrobial activity of the films could be enhanced by increasing the concentration of SFCO or optimizing the film formulation to improve its release and efficacy against microorganisms.

## 4. Conclusions

This study demonstrates the promising potential of biodegradable PVA films loaded with silk fibroin functionalized with copaiba oleoresin (SFCO) as an innovative material for food packaging applications. The developed films exhibit significant improvements in physicochemical, nanomorphological, and antimicrobial properties, which are critical for extending the shelf life of perishable food products. The incorporation of SFCO not only enhanced the mechanical strength of the films but also significantly improved their barrier properties, making them effective in preventing moisture loss and preserving the quality of packaged foods. The films exhibited superior antimicrobial activity, particularly against *Staphylococcus aureus* and *Escherichia coli*, attributed to the bioactive compounds in copaiba oil. This dual functionality of the films, acting both as a physical barrier and as an active antimicrobial agent, positions them as a viable eco-friendly alternative to conventional plastic packaging. Furthermore, the nanomorphological analysis revealed a well-distributed roughness on the film surfaces, which can be tailored by adjusting the concentration of SFCO to meet specific application requirements. The film’s ability to encapsulate and gradually release the bioactive components of copaiba oil underscores its potential for use in applications where the controlled release of antimicrobial agents is desired. Overall, the findings provide a strong foundation for the development of next-generation bioactive packaging materials that align with the growing demand for sustainable, biodegradable solutions in the food industry. The successful integration of silk fibroin and copaiba oleoresin into PVA films not only enhances their functional properties but also contributes to environmental sustainability, offering a significant advancement in the field of polymeric biomaterials for food packaging.

## Figures and Tables

**Table 1:** Formulations of SFCO-loaded PVA films.

Formulation	PVA (% *w*/*v* H_2_O)	SFCO (% *w*/*w* PVA)
Blank–PVA5	5	0
PVA5–SFCO5	5	5
PVA5–SFCO10	5	10
PVA10–SFCO5	10	5
PVA10–SFCO10	10	10

**Table 2:** Mechanical and interfacial properties: contact angle (θ), hygroscopicity (HG), solubility (SL), water vapor transmissionrate (WVTR), Young’s modulus (YM), and elongation at break (EB) of SF/CO-loaded PVA films. Different letters in the same column indicate significant differences between samples with *p* < 0.05.

Sample	θ (°)	HG (%)	SL (%)	WVTR (g/h∙m^2^)	YM (MPa)	EB (%)
Blank–PVA5	72.3 ± 0.06 ^a^	193.43 ± 0.28 ^a^	3.670 ± 0.083 ^a^	101.44 ± 1.18 ^a^	41.65 ± 3.80 ^a^	314.03 ± 9.69 ^a^
PVA5–SFCO5	26.2 ± 0.30 ^b^	173.17 ± 1.01 ^b^	3.072 ± 0.100 ^b^	80.21 ± 1.18 ^a^	57.33± 7.34 ^b^	336.45 ± 38.97 ^a^
PVA5–SFCO10	42.6 ± 0.30 ^c^	161.77 ± 0.31 ^c^	2.305 ± 0.021 ^c^	25.95 ± 1.18 ^b^	139.70 ± 41.80 ^c^	288.90 ± 19.34 ^a^
PVA10–SFCO5	27.4 ± 0.10 ^e^	140.19 ± 1.54 ^e^	0.724 ± 0.073 ^e^	62.51 ± 1.18 ^d^	136.87 ± 12.17 ^c^	288.09 ± 40.54 ^a^
PVA10–SFCO10	34.6 ± 0.23 ^d^	108.58 ± 1.48 ^d^	0.030 ± 0.001 ^d^	37.74 ± 1.18 ^c^	144.59 ± 14.69 ^c^	385.21 ± 68.91 ^a^

Equal letters in the same column indicate statistically equal means, according to ANOVA and Tukey’s test (*p* < 0.05).

**Table 3:** Measurements derived from AFM force spectroscopy.

Sample	Adhesion Force (nN)	Deformation (nm)	Elastic Modulus (GPa)
Blank–PVA5	356.7 ± 0.2 ^a^	5.9 ± 0.003 ^a^	17.0 ± 0.01 ^a^
PVA5–SFCO5	212.6 ± 0.2 ^b^	4.2 ± 0.02 ^b^	22.7 ± 0.02 ^b^
PVA5–SFCO10	251.6 ± 0.2 ^c^	3.8 ± 0.01 ^c^	17.7 ± 0.02 ^a^
PVA10–SFCO5	327.2 ± 0.2 ^d^	3.6 ± 0.002 ^c^	14.4 ± 0.01 ^c^
PVA10–SFCO10	299.7 ± 0.2 ^e^	3.05 ± 0.002 ^d^	22.08 ± 0.02 ^b^

Equal letters in the same column indicate statistically equal means according to ANOVA and Tukey’s test (*p* < 0.05).

**Table 4:** Key roughness and height parameters derived from AFM images. The acronyms are defined as follows: RMS (root mean square roughness), L (lateral correlation length), and E_T_ (topographical entropy).

Sample	RMS (nm)	L (µm)	E_T_ (−)
Blank–PVA5	9.14 ± 0.67 ^a^	1.79 ± 0.34 ^a^	0.924 ± 0.011 ^a^
PVA5–SFCO5	2.70 ± 0.28 ^b^	0.40 ± 0.05 ^b^	0.968 ± 0.005 ^a^
PVA5–SFCO10	11.52 ± 0.64 ^c^	1.57 ± 0.37 ^a^	0.972 ± 0.011 ^a^
PVA10–SFCO5	5.73 ± 0.59 ^d^	2.00 ± 0.41 ^a^	0.943 ± 0.015 ^a^
PVA10–SFCO10	9.10 ± 1.64 ^a^	1.04 ± 0.21 ^c^	0.872 ± 0.027 ^a^

Equal letters in the same column indicate statistically equal means according to ANOVA and Tukey’s test (*p* < 0.05).

**Table 5:** Comparison of the antimicrobial activity zone of the PVA films containing SF-CO solution with free copaiba oil (CO) and gentamicin control.

Sample	*S. aureus* (mm)	*E. coli* (mm)
PVA5–SFCO5	8 ± 1 ^a^	8 ± 1 ^ad^
PVA5–SFCO10	9 ± 1 ^a^	8 ± 1 ^ad^
PVA10–SFCO5	10 ± 1 ^ad^	8 ± 1 ^ad^
PVA10–SFCO10	8 ± 1 ^a^	8 ± 1 ^ad^
Gentamicin	16 ± 1 ^bc^	15 ± 1 ^b^
CO	14 ± 1 ^cd^	12 ± 1 ^cd^
SFCO	12 ± 1 ^d^	10 ± 1 ^d^

Equal letters in the same column indicate statistically equal means, according to ANOVA and Tukey’s test (*p* < 0.05).

**Table 6:** Evaluation of the minimum inhibitory concentration (MIC) of the samples, expressed in µg.mL^−1^.

Sample	*S. aureus* (µg.mL^−1^)	*E. coli* (µg.mL^−1^)
CO	50	50
SFCO	50	100
PVA5–SFCO5	100	100
PVA5–SFCO10	100	100
PVA10–SFCO5	100	100
PVA10–SFCO10	100	100

## Data Availability

The data used to support the findings of this study are available from the corresponding authors upon request.

## References

[1] Vieira A.C.P., Buainain A.M., Spers E.E. (2010). A Segurança Do Alimento e a Necessidade Da Informação Aos Consumidores. Cad. Direito.

[2] Rosseto M., Rigueto C.V.T., Dettmer A., Loss R.A., Pizzutti I.R., Richards N.S.P.D.S. (2021). Adição de Compostos Bioativos Em Embalagens Alimentícias Ativas e Inteligentes: Tendências, Avanços e Desafios. Compostos Bioativos e Suas Aplicações.

[3] Victoria F.N. (2021). A Utilização de Compostos Bioativos Em Embalagens Ativas Como Uma Estratégia Sustentável. Compostos Bioativos e Suas Aplicações.

[4] Vermeiren L., Devlieghere F., Debevere J. (2002). Effectiveness of Some Recent Antimicrobial Packaging Concepts. Food Addit. Contam..

[5] Hernández-García E., Vargas M., González-Martínez C., Chiralt A. (2021). Biodegradable Antimicrobial Films for Food Packaging: Effect of Antimicrobials on Degradation. Foods.

[6] Estevão L.R.M., Medeiros J.P.d., Baratella-Evêncio L., Simões R.S., Mendonça F.d.S., Evêncio-Neto J. (2013). Effects of the Topical Administration of Copaiba Oil Ointment (*Copaifera Langsdorffii*) in Skin Flaps Viability of Rats. Acta Cir. Bras..

[7] Gelmini F., Beretta G., Anselmi C., Centini M., Magni P., Ruscica M., Cavalchini A., Maffei Facino R. (2013). GC–MS Profiling of the Phytochemical Constituents of the Oleoresin from Copaifera Langsdorffii Desf. and a Preliminary in Vivo Evaluation of Its Antipsoriatic Effect. Int. J. Pharm..

[8] Morelli C.L., Mahrous M., Belgacem M.N., Branciforti M.C., Bretas R.E.S., Bras J. (2015). Natural Copaiba Oil as Antibacterial Agent for Bio-Based Active Packaging. Ind. Crops Prod..

[9] Paiva L.A.F., de Alencar Cunha K.M., Santos F.A., Gramosa N.V., Silveira E.R., Rao V.S.N. (2002). Investigation on the Wound Healing Activity of Oleo-resin from Copaifera Langsdorffi in Rats. Phyther. Res..

[10] Souza Barbosa P.C., Moreira Wiedemann L.S., da Silva Medeiros R., de Tarso Barbosa Sampaio P., Vieira G., Florêncio da Veiga-Junior V. (2013). Phytochemical Fingerprints of Copaiba Oils (*Copaifera Multijuga* Hayne) Determined by Multivariate Analysis. Chem. Biodivers..

[11] Lemos M., Santin J.R., Mizuno C.S., Boeing T., de Sousa J.P.B., Nanayakkara D., Bastos J.K., de Andrade S.F. (2015). Copaifera Langsdorffii: Evaluation of Potential Gastroprotective of Extract and Isolated Compounds Obtained from Leaves. Rev. Bras. Farmacogn..

[12] Santiago K.B., Conti B.J., Murbach Teles Andrade B.F., Mangabeira da Silva J.J., Rogez H.L.G., Crevelin E.J., Beraldo de Moraes L.A., Veneziani R., Ambrósio S.R., Bastos J.K. (2015). Immunomodulatory Action of Copaifera Spp Oleoresins on Cytokine Production by Human Monocytes. Biomed. Pharmacother..

[13] Lima S.R.M., Junior V.F.V., Christo H.B., Pinto A.C., Fernandes P.D. (2003). In Vivo and in Vitro Studies on the Anticancer Activity of Copaifera Multijuga Hayne and Its Fractions. Phyther. Res..

[14] Mustapa A.N., Martin A., Sanz-Moral L.M., Rueda M., Cocero M.J. (2016). Impregnation of Medicinal Plant Phytochemical Compounds into Silica and Alginate Aerogels. J. Supercrit. Fluids.

[15] Ferreira I.M., Nishimura R.H.V., Souza A.B.d.A., Clososki G.C., Yoshioka S.A., Porto A.L.M. (2014). Highly Enantioselective Acylation of Chlorohydrins Using Amano AK Lipase from P. Fluorescens Immobilized on Silk Fibroin–alginate Spheres. Tetrahedron Lett..

[16] Wang Z., Cui Y., Feng Y., Guan L., Dong M., Liu Z., Liu L. (2021). A Versatile Silk Fibroin Based Filtration Membrane with Enhanced Mechanical Property, Disinfection and Biodegradability. Chem. Eng. J..

[17] Li F., Wang X., Chen L., Li Z., Zhang T., Wang T. (2021). Efficient Development of Silk Fibroin Membranes on Liquid Surface for Potential Use in Biomedical Materials. Int. J. Biol. Macromol..

[18] Jin H.-J., Kaplan D.L. (2003). Mechanism of Silk Processing in Insects and Spiders. Nature.

[19] Bini E., Knight D.P., Kaplan D.L. (2004). Mapping Domain Structures in Silks from Insects and Spiders Related to Protein Assembly. J. Mol. Biol..

[20] Tavares-Dias M., Neves L.R., Alves C.M.G., Nogueira J.N., Neves F.B., Pinto A.V.P., Carvalho J.C.T., Ferreira I.M. (2021). In Vitro Anthelminthic Activity of Lippia Alba Essential Oil Combined with Silk Fibroin against Monogeneans of Colossoma Macropomum (Serrasalmidae). Aquac. Res..

[21] Echeverría J., Albuquerque R. (2019). Nanoemulsions of Essential Oils: New Tool for Control of Vector-Borne Diseases and In Vitro Effects on Some Parasitic Agents. Medicines.

[22] Ferreira I.M., Ganzeli L.d.S., Rosset I.G., Yoshioka S.A., Porto A.L.M. (2017). Ethylic Biodiesel Production Using Lipase Immobilized in Silk Fibroin-Alginate Spheres by Encapsulation. Catal. Letters.

[23] Jain N., Singh V.K., Chauhan S. (2017). A Review on Mechanical and Water Absorption Properties of Polyvinyl Alcohol Based Composites/Films. J. Mech. Behav. Mater..

[24] Limpan N., Prodpran T., Benjakul S., Prasarpran S. (2012). Influences of Degree of Hydrolysis and Molecular Weight of Poly(Vinyl Alcohol) (PVA) on Properties of Fish Myofibrillar Protein/PVA Blend Films. Food Hydrocoll..

[25] Pérez-Córdoba L.J., Sánchez-Pizarro A., Vélez-Erazo E.M., Peña-Carrasco E.F., Pasquel-Reátegui J.L., Martínez-Tapia P., Velezmoro-Sánchez C. (2024). Bitter Potato Starch-based Film Enriched with Copaiba Leaf Extract Obtained Using Supercritical Carbon Dioxide: Physical–mechanical, Antioxidant, and Disintegrability Properties. J. Appl. Polym. Sci..

[26] Silva I.D.L. (2020). Desenvolvimento de Filmes Ativos Antimicrobianos e Antioxidantes de PVA Aditivado com Extratos Vegetais de Plantas do Sertão. Ph.D. Thesis.

[27] de Carvalho S.M., Noronha C.M., da Rosa C.G., Sganzerla W.G., Bellettini I.C., Nunes M.R., Bertoldi F.C., Manique Barreto P.L. (2019). PVA Antioxidant Nanocomposite Films Functionalized with Alpha-Tocopherol Loaded Solid Lipid Nanoparticles. Colloids Surfaces A Physicochem. Eng. Asp..

[28] Schindelin J., Arganda-Carreras I., Frise E., Kaynig V., Longair M., Pietzsch T., Preibisch S., Rueden C., Saalfeld S., Schmid B. (2012). Fiji: An Open-Source Platform for Biological-Image Analysis. Nat. Methods.

[29] Sun X., Yin L., Zhu H., Zhu J., Hu J., Luo X., Huang H., Fu Y. (2022). Enhanced Antimicrobial Cellulose/Chitosan/ZnO Biodegradable Composite Membrane. Membranes.

[30] (2012). Test Methods for Water Vapor Transmission of Materials.

[31] Scatolino M.V., Bufalino L., Dias M.C., Mendes L.M., da Silva M.S., Tonoli G.H.D., de Souza T.M., Junior F.T.A. (2022). Copaiba Oil and Vegetal Tannin as Functionalizing Agents for Açai Nanofibril Films: Valorization of Forest Wastes from Amazonia. Environ. Sci. Pollut. Res..

[32] (2002). Standard Test Method for Tensile Properties of Thin Plastic Sheeting.

[33] Nečas D., Klapetek P. (2012). Gwyddion: An Open-Source Software for SPM Data Analysis. Cent. Eur. J. Phys..

[34] Matos R.S., Lopes G.A.C., Ferreira N.S., Pinto E.P., Carvalho J.C.T., Figueiredo S.S., Oliveira A.F., Zamora R.R.M. (2018). Superficial Characterization of Kefir Biofilms Associated with Açaí and Cupuaçu Extracts. Arab. J. Sci. Eng..

[35] Nečas D., Klapetek P. (2013). One-Dimensional Autocorrelation and Power Spectrum Density Functions of Irregular Regions. Ultramicroscopy.

[36] Pinto E.P., Pires M.A., Matos R.S., Zamora R.R.M., Menezes R.P., Araújo R.S., de Souza T.M. (2021). Lacunarity Exponent and Moran Index: A Complementary Methodology to Analyze AFM Images and Its Application to Chitosan Films. Phys. A Stat. Mech. Its Appl..

[37] Racine J.S. (2012). RStudio: A Platform-Independent IDE for R and Sweave. J. Appl. Econom..

[38] Ramos G.Q., Melo I.d.C., Matos R.S., Pinto E.P., Pires M.A., Sanches E.A., Filho H.D.d.F. (2023). SEM-Imaging-Based Mapping of Monofractal and Multifractal Patterns of the Piper Krukoffii Yunck Leaf Surface Architecture. Flora.

[39] Ţălu Ş., Stach S., Ghodselahi T., Ghaderi A., Solaymani S., Boochani A., Garczyk Ż. (2015). Topographic Characterization of Cu–Ni NPs @ A-C:H Films by AFM and Multifractal Analysis. J. Phys. Chem. B.

[40] Bauer A.W. (1966). Antibiotic Susceptibility Testing by a Standardized Single Disc Method. Am. J. Clin. Pathol..

[41] Hammer D.A.T., Ryan P.D., Hammer Ø., Harper D.A.T. (2001). Past: Paleontological Statistics Software Package for Educaton and Data Anlysis. Palaeontol. Electron..

[42] Sionkowska A., Planecka A. (2011). The Influence of UV Radiation on Silk Fibroin. Polym. Degrad. Stab..

[43] Koperska M.A., Pawcenis D., Bagniuk J., Zaitz M.M., Missori M., Łojewski T., Łojewska J. (2014). Degradation Markers of Fibroin in Silk through Infrared Spectroscopy. Polym. Degrad. Stab..

[44] Tsuboi Y., Ikejiri T., Shiga S., Yamada K., Itaya A. (2001). Light Can Transform the Secondary Structure of Silk Protein. Appl. Phys. A Mater. Sci. Process..

[45] Nogueira R.J.L., Grazul R.M., Silva Filho A.A.D., Nascimento J.W.L. (2022). Evaluation of Copaiba Oil as Enhancer of Ibuprofen Skin Permeation. Brazilian J. Pharm. Sci..

[46] Chen Z., Zong L., Chen C., Xie J. (2020). Development and Characterization of PVA-Starch Active Films Incorporated with β-Cyclodextrin Inclusion Complex Embedding Lemongrass (Cymbopogon Citratus) Oil. Food Packag. Shelf Life.

[47] Alizadeh-Sani M., Rhim J.-W., Azizi-Lalabadi M., Hemmati-Dinarvand M., Ehsani A. (2020). Preparation and Characterization of Functional Sodium Caseinate/Guar Gum/TiO2/Cumin Essential Oil Composite Film. Int. J. Biol. Macromol..

[48] Sapper M., Wilcaso P., Santamarina M.P., Roselló J., Chiralt A. (2018). Antifungal and Functional Properties of Starch-Gellan Films Containing Thyme (Thymus Zygis) Essential Oil. Food Control.

[49] Alarcón-Moyano J.K., Bustos R.O., Herrera M.L., Matiacevich S.B. (2017). Alginate Edible Films Containing Microencapsulated Lemongrass Oil or Citral: Effect of Encapsulating Agent and Storage Time on Physical and Antimicrobial Properties. J. Food Sci. Technol..

[50] Kaewpirom S., Piboonnithikasem S., Sroisroemsap P., Uttoom S., Boonsang S. (2024). Tailoring Silk Fibroin Hydrophilicity and Physicochemical Properties Using Sugar Alcohols for Medical Device Coatings. Sci. Rep..

[51] Li T.-T., Zhong Y., Peng H.-K., Ren H.-T., Chen H., Lin J.-H., Lou C.-W. (2021). Multiscale Composite Nanofiber Membranes with Asymmetric Wetability: Preparation, Characterization, and Applications in Wound Dressings. J. Mater. Sci..

[52] Mejía Suaza M.L., Leos Rivera J.C., Rodríguez Padilla M.C., Moncada Acevedo M.E., Ossa Orozco C.P., Zarate Triviño D.G. (2023). Poly(Vinyl Alcohol)/Silk Fibroin/Ag-NPs Composite Nanofibers as a Substrate for MG-63 Cells’ Growth. Polymers.

[53] Sau S., Pandit S., Kundu S. (2021). Crosslinked Poly (Vinyl Alcohol): Structural, Optical and Mechanical Properties. Surf. Interfaces.

[54] Abbott E.J., Firestone F.A. (1933). Specifying Surface Quality. Mech. Eng..

[55] Schmähling J., Hamprecht F.A. (2007). Generalizing the Abbott–Firestone Curve by Two New Surface Descriptors. Wear.

[56] Dzyura V., Maruschak P., Slavov S., Dimitrov D., Semehen V., Markov O. (2023). Evaluating Some Functional Properties of Surfaces with Partially Regular Microreliefs Formed by Ball-Burnishing. Machines.

[57] Zhang Y., Sundararajan S. (2005). The Effect of Autocorrelation Length on the Real Area of Contact and Friction Behavior of Rough Surfaces. J. Appl. Phys..

[58] Matos R.S., Ferreira N.S., Ţălu Ş., Ghaderi A., Solaymani S., Pires M.A., Sanches E.A., da Fonseca Filho H.D. (2022). Percolative, Multifractal, and Symmetry Properties of the Surface at Nanoscale of Cu-Ni Bimetallic Thin Films Deposited by RF-PECVD. Symmetry.

[59] Sadeghi M., Zelati A., Rezaee S., Luna C., Matos R., Pires M., Ferreira N., da Fonseca Filho H., Ahmadpourian A., Ţălu Ş. (2022). Evaluating the Topological Surface Properties of Cu/Cr Thin Films Using 3D Atomic Force Microscopy Topographical Maps. Coatings.

[60] Ramos G.Q., Matos R.S., Das A., Kumar S., Ţălu Ş., da Fonseca Filho H.D. (2022). Correlating Morphology and Multifractal Spatial Patterns of the Leaf Surface Architecture of *Anacardium occidentale* L.. Fractal Fract..

[61] Chai C., Lee K.-S., Oh S.-W. (2015). Synergistic Inhibition of Clostridium Difficile with Nisin-Lysozyme Combination Treatment. Anaerobe.

[62] Nostro A., Scaffaro R., D’Arrigo M., Botta L., Filocamo A., Marino A., Bisignano G. (2012). Study on Carvacrol and Cinnamaldehyde Polymeric Films: Mechanical Properties, Release Kinetics and Antibacterial and Antibiofilm Activities. Appl. Microbiol. Biotechnol..

[63] Laurenti M., Cauda V. (2018). Gentamicin-Releasing Mesoporous ZnO Structures. Materials.

[64] Pormohammad A., Hansen D., Turner R.J. (2022). Antibacterial, Antibiofilm, and Antioxidant Activity of 15 Different Plant-Based Natural Compounds in Comparison with Ciprofloxacin and Gentamicin. Antibiotics.

[65] Tobouti P.L., de Andrade Martins T.C., Pereira T.J., Mussi M.C.M. (2017). Antimicrobial Activity of Copaiba Oil: A Review and a Call for Further Research. Biomed. Pharmacother..

[66] Alencar É.N., Xavier-Júnior F.H., Morais A.R.V., Dantas T.R.F., Dantas-Santos N., Verissimo L.M., Rehder V.L.G., Chaves G.M., Oliveira A.G., Egito E.S.T. (2015). Chemical Characterization and Antimicrobial Activity Evaluation of Natural Oil Nanostructured Emulsions. J. Nanosci. Nanotechnol..

[67] Santos S.B. (2021). dos Modulação Antimicrobiana Do Extrato Da Entrecasca Libidibiaferrea. Int. J. Dev. Res..

